# Risk Factors of Suicide Death Based on Psychological Autopsy Method; a Case-Control Study 

**Published:** 2019-09-01

**Authors:** Nafee Rasouli, Seyed Kazem Malakouti, Mohsen Rezaeian, Seyed Mehdi Saberi, Marzieh Nojomi, Diego De Leo, Abbas Ramezani-Farani

**Affiliations:** 1 Mental Health Research Center, School of Behavioral Sciences and Mental Health (Tehran Institute of Psychiatry), Iran University of Medical Sciences, Tehran, Iran.; 2 Epidemiology and Biostatistics Department, Rafsanjan Medical School, Occupational Environmental Research Center, Rafsanjan University of Medical Sciences, Rafsanjan, Iran.; 3 Legal Medicine Research Center, Legal Medicine Organization, Tehran, Iran.; 4 Preventive Medicine and Public Health Research Center, Psychosocial Health Research Institute, Department of Community and Family Medicine, Iran University of Medical Sciences, Tehran, Iran.; 5 Australian Institute for Suicide Research and Prevention, Griffith University, Brisbane, Queensland, Australia.; 6 Department of Clinical Psychology, School of Behavioral Sciences and Mental Health (Tehran Institute of Psychiatry), Iran University of Medical Sciences, Tehran, Iran.

**Keywords:** Suicide, risk factors, mental disorders, depression, psychological autopsy

## Abstract

**Introduction:**

Investigation in each community can contribute to understanding the key factors involved in suicide death and its prevention. The present study aimed to investigate suicide death risk factors based on psychological autopsy method.

**Methods:**

The present case-control study was conducted from April to September 2017, in Tehran, Iran, to compare two groups of people; those who died by suicide and controls (over the age of 18 years). Data were collected by one interviewer via Structured Clinical Interviews (SCID-I), questionnaires used in the SUPREMISS study, and the Dickman impulsivity scale.

**Results:**

Each group consisted of 40 individuals. There was no significant difference between the case and control groups in terms of all demographic variables except for the level of education (p = 0.06) and occupational status (p = 0.009). The frequency of previous history of suicide attempt (p = 0.001), family history of suicide (p = 0.003), DSM IV Axis I disorders (p = 0.006), and substance and alcohol consumption (p = 0.01) were significantly higher in the case group. The most commonly diagnosed disorders included MDD (45%) and substance use disorders (30%), respectively. The most common methods used in suicide included hanging (32.5%), and Aluminum phosphide poisoning (32.5%) known as rice tablet. The strongest predictor of suicide death was the deceased person's Previous history of suicide attempt (OR= 9.3; p = 0.04), smoking (OR= 6.4; p = 0.006), unemployment (OR= 5; p = 0.02), and DSM IV axis I disorders (OR= 3.8; p = 0.04).

**Conclusion:**

Previous suicide attempt, smoking, unemployment, and suffering from at least one mental disorder were the significant predictors of suicide death. Among mental disorders, major depressive disorder and substance use disorder were the most prevalent mental health problems.

## Introduction

One person ends his/her life through suicide somewhere in the world every 40 seconds; however, suicide is preventable. At least 6 people are directly affected by any suicide-related death (1). Since suicide is a very sensitive issue, and even illegal in some countries (2, 3), it is likely to be reported less than the actual rate. An increasing trend in suicide rate has been revealed in Iran through research in the last two decades (4). Suicides and attempted suicides have increased from 8.3 per 100,000 population in 2001 to 19.4 in 2005 (5, 6). The stated figure is smaller than most local reports that have been carried out as research studies (6); however, there is great variation in the prevalence of suicide in different regions of the country. Western provinces of Iran had the highest rate of suicide, from 10 to 20 suicide cases, per 100,000 people between 2006 and 2010. In 2012, the suicide rate per 100,000 people was 5.3 in both sexes, 3.6 in females, and 7.0 in males (1). Each day, more than 13 people take their own lives by suicide in Iran; most of whom are aged 15-35 years. The average nationwide rate of suicide has risen from 4.25 to 4.88 (from 2006 to 2010) and from 5.4 to 5.8 (2012-2017) according to the Legal Medicine Organization (LMO) (7).

Psychiatric disorders are considered very important risk factors affecting suicidal behavior and earlier psychological autopsy (PA) studies have reported a strong relationship between mental disorders and suicide deaths. Previous studies in developed countries showed that 60-90% of individuals who died by suicide had been suffering from at least one mental disorder (8, 9), with mood disorders being considered as the most commonly diagnosed conditions in most studies (10, 11). In addition, the prevalence of mental disorders varies based on gender in such a way that the prevalence of substance use and personality disorders were significantly higher in males, but mood disorders, especially major depressive disorder (MDD) were more common in females (8).

Psychological autopsy is a process that involves rewriting the deceased's biography through psychological information extracted from personal records such as police records, medical records and interviews with family, friends, co-workers, and physicians. Today, the psychological autopsy method has broader applications including the less ambiguous suicide death cases. Risk factors, personality of the deceased person, and suicidal intentions and motives are investigated using different methods such as family interviews, suicide notes, etc. (12). Psychological autopsy studies using a strong methodology have not been carried out in Iran; therefore, investigating risk factors of suicide death cases in Iran can help achieve a better understanding of these factors and be effective in preventing future suicide attempts. 

**Table 1:** Baseline characteristics of studied subjects

**P value**	**Control (n=40) **	**Case (n=40) **	**Variables**
			**Gender**
0.2	30 (75.0)	32 (80.0)	Male
	10 (25.0)	8 (20.0)	Female
			**Age (years) **
0.9	37.23 ±13.7	39.42 ±16.06	Mean ± SD
			**Education **
0.06	26 (65)	18 (45)	High school diploma and higher
	14 (35)	22 (55)	Less than high school diploma
			**Marital status **
0.2	18 (45)	15 (37.5)	Single
	21 (52.5)	20 (50)	Married
	1 (2.5)	5 (12.5)	Divorced
			**Living arrangement **
0.2	2 (5.0)	4 (10.0)	Lived alone
	34 (85.0)	35 (87.5)	With family members
	4 (10.0)	1 (2.5)	With others
			**Occupational status**
0.009	7 (17.5)	24 (60.0)	Unemployed
	33 (82.5)	16 (40.0)	Employed
			**SCID-I disorder **
0.006	30 (75.0)	7 (17.5)	No
	10 (25.0)	33 (82.5)	Yes
			**History**
0.001	1 (2.5)	17 (42.5)	Previous suicide attempt
0.003	-	7 (17.5)	Family history of suicide attempt
0.01	5 (12.5)	15 (37.5)	History of substance and alcohol consumption
0.001	10 (25)	30 (75)	Tobacco use
			**Suicide method **
NA	-	13 (32.5)	Hanging
	-	13 (32.5)	Aluminum phosphide poisoning
	-	6 (15)	Jumping from a height
	-	5 (12.5)	Self-injury
	-	1 (2.5)	Burning
	-	1 (2.5)	Acid intake
	-	1 (2.5)	Carbon monoxide poisoning
			**Drugs/substance abuse**
	-	4 (10.0)	Tranquillizers
	-	9 (22.5)	Opioid
	-	2 (5.0)	Cannabis
	-	7 (17.5)	Crystal
	-	1 (2.5)	Other

Data are presented as mean ± standard deviation (SD) or frequency (%). SCID: The Structured Clinical Interview for DSM-IV Axis; NA: not applicable.

**Table 2:** Case-control comparisons of mental disorders, anger, and impulsivity

**Variables**	**Case **	**Control**	**P value**
Major depressive disorder	18 (45.0)	2 (5.0)	0.003
Bipolar disorders	6 (15)	1 (2.5)	0.04
Anxiety disorders	7 (17.5)	7 (17.5)	1
Psychotic disorders	6 (15.0)	-	0.01
Substance-related disorders	12 (30.0)	-	0.001
SCID-I disorder**	33 (82.5)	10 (25.0)	0.006
Anger^# ^	15.6 ± 8.4	9.9 ± 5.8	0.1
Impulsivity*	9.8 ± 5.5	7.6 ± 3.9	0.2

Data are presented as mean ± standard deviation (SD) or frequency (%). *: Based on Dickman impulsivity scale, #: based on Spielberger's State-Trait Anger Scale (STAS);**The Structured Clinical Interview for DSM-IV Axis I (SCID).

**Table 3:** Risk factors of suicide based on logistic regression model

**P value**	**OR (95% CI)**	** df**	**Wald**	S.E.*	** B**	**Variables**
0.04	3.8 (1.06 -13.9)	1	4.2	0.6	1.3	SCID-I disorder
0.02	5 (1.2 – 20.2)	1	5.1	0.7	1.6	Unemployment
0.006	6.4 (1.7 – 24.5)	1	7.5	0.6	1.8	Tobacco use
0.04	9.3 (1.03 – 83.7)	1	3.9	1.1	2.2	Previous suicide attempt

*SE: standard error; OR: odds ratio; CI: confidence interval; SCID: The Structured Clinical Interview for DSM-IV Axis. Each of the variables predicting the suicide was reported at ≤0.05 based on the significance level

## Methods

***Study design and setting***

The present case-control study was conducted from April to September 2017 to compare two groups of people, those who died by suicide and the control group. The sample size consisted of 40 individuals for each group. Data were collected by one interviewer via Structured Clinical Interviews (SCID-I), questionnaires used in the SUPREMISS study, and the Dickman impulsivity scale. The protocol of study was approved by the Ethics committee of Iran University of Medical Sciences (ethical code: IR.IUMS.FMD.REC1396.9411).

***Participants***

The case group consisted of individuals over the age of 18 years, who died by suicide in Tehran, the capital of Iran. The controls were alive subjects homogenous with the suicide group in terms of age, gender, and place of residence.

The study was performed initially by accessing the medical records of the deceased for six months from April to September 2017, through coordination with the Legal Medicine Organization (LMO) of Tehran. Around 800 medical records susceptible to suicide were introduced to the investigator, which were scrutinized carefully considering inclusion and exclusion criteria. Inclusion criteria included: 1) Individuals who died by suicide at least 3 months before the study, 2) people over 18 years of age, 3) lack of ambiguity regarding the suicide cause of death as recorded in the medical records. The interval of 3 months was considered due to the following reasons: first, the mourning period has been partially passed and, second, there is lower risk of memory distortion in survivors within 3 months. Using purposive and non-randomized sampling, eligible cases were contacted by telephone call (220 close relatives and families).

The research objectives and manner of accessing the information were explained to each of the close relatives and families who accepted to take part in the study, via telephone contact. If they were willing to do so, face-to-face interview was arranged at the participant's home. Each interview lasted 2 hours, on average. The interviewees consisted of first-degree family members, including 22 parents (18 of whom were mothers), 2 sons aged above 30 years, 8 spouses and 5 sisters, and 3 brothers, who were well aware of the symptoms and other characteristics of the deceased. 

The control subjects were selected purposively and non-randomly, and consisted of 30 males and 10 females who were selected from the accessible individuals who were living in Tehran trying to match cases by gender and age. Inclusion criteria were 1) Not having history of suicide attempt; 2) Being willing to cooperate in the project. 

***Data gathering Instruments***

SUPREMISS study protocol was used for collecting the data in this study. This protocol includes a set of input questionnaire (items 1-14), demographic questionnaire (15-27), questionnaire on history of previous suicide attempt (s) and suicide ideation in the family (28-40), anger scale (56-65), alcohol and drug use questionnaire (66-77). 

**- Spielberger's State-Trait Anger Scale (STAS)**

This scale measures the severity of anger and includes 10 items. Questionnaire on Anger was developed in 1980. This is a self-report instrument designed in the 1970s. This instrument has been validated in Iran in various studies including those carried out by Khodayarifard et al., in which the internal consistency and test-retest reliability coefficient of the above instrument were reported to be 0.60-0.93 and 0.72-0.93 (13).

**- Dickman Impulsivity questionnaire**

This questionnaire was validated by Ekhtiari et al. (2008) in Iran. The internal consistency of Cronbach's alpha was 0.69 in healthy subjects and 0.74 in substance abusers. The Cronbach's alpha of dysfunctional impulsivity subscale was 0.48 in healthy subjects and 0.42 in substance abusers (14).

**- The Structured Clinical Interview for DSM-IV Axis I (SCID-I)**

This provides a clinician administered semi structured scripted approach for evaluation of psychopathology and to determine current or lifetime presence of psychiatric diagnoses according to DSM-IV Criteria (15). The diagnostic agreement for most of the specific and general diagnoses ranged from moderate to good (kappa coefficient higher than 0.6). In one study, the total kappa coefficient was reported to be 0.55 and 0.52 for lifetime and current diagnoses, respectively (16).

***Statistical analysis***

Considering the minimum difference in the frequency of mental disorders in the suicide group (90%) with the control group (30%), based on the findings of Cavanagh et al.'s (9) meta-analysis, and type I error=5% and power= 90%, sample size was determined as 20 cases. Chi-square and T-test were used to compare qualitative and quantitative variables between the two groups, respectively. Variables that were significantly different between the two groups were placed in the regression equation. Some variables that had very high confidence interval were excluded from the regression analysis since they were significantly different between the two groups.

## Results

***Baseline characteristics of participants***

The suicide group consisted of 40 people (80% male; mean age = 39.4 ± 16; range =19-75); and the control group also consisted of 40 individuals who were homogeneous with the suicide group (75% male; mean age = 37.2 ± 13.7; range = 19-72). Table 1 shows the baseline characteristics of studied subjects. There was no significant difference between the case and control groups in terms of all demographic variables except for the level of education (p = 0.06) and occupational status (p = 0.009). The frequency of previous history of suicide attempt (p = 0.0010(, family history of suicide (p = 0.003), DSM IV Axis I disorders (p = 0.006), and substance and alcohol consumption (p = 0.01) were significantly higher in the case group. The most commonly diagnosed disorders included MDD (45%) and substance use disorders (30%), respectively. The most common methods used in suicide included hanging (32.5%), and Aluminum phosphide poisoning (32.5%) known as rice tablet. Opioids (22.5%) had the highest prevalence rate among the substances used, which included opium (10%), heroin (7.5%), methadone (5%), and tramadol (7.5%). Table 2 compares the frequency of mental disorders, anger, and impulsivity between case and control groups.

***Predictive factors of suicide***

The results of regression analysis revealed that the strongest predictor of suicide death was the deceased person's Previous history of suicide attempt (OR= 9.3; p = 0.04), smoking (OR= 6.4; p = 0.006), unemployment (OR= 5; p = 0.02), and axis I disorders (OR= 3.8; p = 0.04) (Table 3).

## Discussion

Based on the findings of the present study, previous suicide attempt, smoking, unemployment, and suffering from at least one mental disorder were the significant predictors of suicide death. Among mental disorders, major depressive disorder and substance use disorder were the most prevalent mental health problems. 

The present study is the first PA study of suicide in Iran with case-control methodology and using structured interview with the study samples. According to the demographic data, the percentage of married and divorced people was higher in the suicide group, which is in line with the results of some other research studies (17, 18). According to the results of some previous studies in Iran (19, 20), marital discord and parent-child generation gap, are the most prevalent stressors before attempting suicide. It may be concluded that, unlike some studies conducted in developed countries (21, 22), marriage is not necessarily a protective factor against suicide. In a review study, which included data from 16 provinces of Iran, marital discord and family problems were the most common causes of suicide attempts (23). Nevertheless, in developed countries, the risk of suicide in divorced men is twice that of single or widowers, but marital status was not influential in women in contrast with the finding of our study (24). The results of a study in Japan showed that unemployment and divorce increase the risk of suicide by up to four times (25). However, studies in developed countries revealed that low marital integration was also associated with a higher risk of suicide (26). In Northern Ireland, the risk of suicide in single, divorced, and widowed men was greater and more significant than women (27). It seems that any change in marital status in terms of divorce, and widowhood in the first five-years of marriage can be a risk factor for suicide, which is related to the social, cultural, and family background of the society (28). Regarding education, there was a significant difference between the two groups, which is consistent with previous studies (21, 29); suicide occurs more frequently among individuals with lower level of education and this is applicable in both genders and all age groups (30). Low education can affect the chance of finding a suitable job and income, which has a significant impact on suicide attempts (31). Most suicide attempters have education level of high school diploma, but the education level is lower among those who died by suicide (32).

In terms of family support, in the current study, 87.5% of individuals lived with their family, 10% lived alone, and 2.5% lived with others. Although most of the individuals in the suicide group lived with their families, it seems that the family were not able to provide enough supportive environment to prevent suicide. Previous studies have shown that loss of family support significantly predicts suicide attempts before attempting suicide (33). Therefore, it can be inferred that positive and supportive interpersonal relationships, and not just physically being together as a family, can play a more effective role in preventing suicide attempts.

The present study revealed that the deceased's previous history of suicide was a strong predictor of suicide (OR = 9). Also other studies referred to the history of suicide attempt as a predictor of suicide (OR = 2-24) (9, 29, 34).

The present study also referred to unemployment as a strong predictor of suicide (OR= 5). Nevertheless, unemployment should be considered with other social factors simultaneously, but unemployment can be considered an important factor in suicide attempts, particularly in low-income countries. Results of a study in Iran showed that the Human Development Index has negative correlation with suicide rate (7). Results of a study on suicide attempters revealed that unemployment increases the risk of dying by suicide by 2.5 times (19). Another study (35) also reported that unemployment along with other factors such as disease, economic problems, and poor supportive systems will affect suicidal behavior.

Tobacco use, was significantly higher in the suicide group compared to the control group (OR = 6.4)(19). Results of an autopsy study on suicidal behaviors in smokers (36) showed that more than 70% of deceased subjects were smokers, while smokers made up 50% of the population in the control group. Previous research studies also showed a significant higher risk of suicide among heavy smokers than those who have rarely or never smoked (37), which may be due to the high incidence of other psychiatric disorders, and particularly major depression in smokers. Some studies have shown that smoking cigarette may be a type of self-healing in depressed people (38). This relationship can also be inverse. Also, some studies (39, 40) reported that smoking increases the risk of depression.

In contrast to our expectation, impulsivity and aggression were not a good predictor of fatal suicidal behavior. Many studies have referred to impulsivity as a major risk factor (41, 42). Impulsive suicide attempts are usually seen in young, single, and aggressive type-B individuals. The average age of subjects participating in the present study was 39 years, which may justify such result (43, 44). Results of a study in Ilam, one of the western provinces of Iran, showed that domestic violence and impulsivity could increase the risk of suicide by 1.1 to 3 times (45). García et al. (2005) examined impulsivity in hospitalized patients and concluded that non-impulsive attempts increase the risk of death more than other attempts and are called ‘more fatal’ (46). However, if dangerous methods such as burning are used in impulsive attempts, the fatality rate is 80% (45)(47). In the present study, most of the subjects (around 50%) attempted suicide with prior planning, eleven had suicide notes and eight others had implicitly pointed to their suicide attempt, which could be considered as non-impulsive suicide attempt. This can be another reason for low impulsive and aggressive behavior among the subjects.

Mental disorders are a strong predictor of suicide and the treatment of these disorders can be very important in preventing suicide. The strength of this research was the use of SCID to diagnose the disorders of deceased individuals by interviewing the first-degree relatives who had the closest relationship with them. One uncontrolled psychological autopsy study carried out in Lorestan province, with high rate of suicide in Iran, showed that 85% of the individuals had mental disorders at the time of death (48). Results of a longitudinal study in Ilam province, south western Iran with high rate of suicide, showed that the rate of suicide caused by psychiatric diseases has increased from 17% to 26% over the past 16 years (49). Our study showed that 82.5% of the deceased people suffered from axis I disorders, while such disorder was prevalent among 25% of the individuals in the control group. The results of other studies indicate that a high percentage (70.4%-88.6%) of the suicide cases had at least one axis I disorder (8, 11, 50). The present study showed that major depression and substance use disorders were the most prevalent disorders among the deceased (with prevalence of 45% and 30%, respectively), which is in line with some other studies (51, 52). A review study (8), which focused on high-income countries, reported that 87% of the suicide cases had at least one axis I disorder. However, this finding is not in line with some Asian countries such as Japan (65%, 72%), India (42%), Hong Kong (69%) and China (48%,63%) (10, 36, 50, 51, 53, 54); but, it is similar to Taiwan (97% -100%) (55) and Pakistan (96%) (56). 

The combination of multi-dimensional risk factors including suffering from mental disorder, unemployment, and smoking may cause a particular group of people with low education, married/divorced, with history of suicide attempt to end their own life by suicide. Regarding the contribution of multiple risk factors in suicide, a comprehensive suicide prevention program is required particularly for geographic regions with high prevalence of suicide.

Conducting psychological autopsies in regions with high rate of suicide in Iran, will make clear understanding of risk factors for suicide in different geographical areas to tailor the national and regional suicide prevention program. 

## Limitations

Obtaining the consent of the deceased's families and interviewing them was one of the challenging issues of conducting this study. Since the present study required a long, in-depth interview, there was a need for participants' consent over their participation in the research and the provision of effective information on the deceased.

## Conclusion:

Based on the findings of the present study, previous suicide attempt, smoking, unemployment, and suffering from at least one mental disorder were the significant predictors of suicide death. Among mental disorders, major depressive disorder and substance use disorder were the most prevalent mental health problems. 
